# Language barriers in pediatric food allergy care: A single-center study on healthcare disparities

**DOI:** 10.1371/journal.pone.0346248

**Published:** 2026-04-01

**Authors:** Katelyn H. Wong, Nishant Pandya, Sarah McCollum, Veronika Shabanova, Gary K. Soffer

**Affiliations:** 1 Department of Pediatrics, Section of Pediatric Pulmonology, Allergy, Immunology and Sleep Medicine, Yale School of Medicine, New Haven, Connecticut, United States of America; 2 Department of Pediatrics, Yale School of Medicine, New Haven, Connecticut, United States of America; 3 Department of Integrative Medicine, Smilow Cancer Center, Yale School of Medicine, New Haven, Connecticut, United States of America; Yonsei University Medical Center: Yonsei University Health System, KOREA, REPUBLIC OF

## Abstract

**Background:**

There is limited knowledge of healthcare utilization and food allergy outcomes among non-primary English-speaking patients in the United States, despite language barriers potentially being a risk factor for less optimal health outcomes.

**Methods:**

A retrospective cohort study was conducted of patients 0−18 years old with food allergy seen at our institution’s allergy and immunology clinic between 1/1/2018-12/31/2021. Only patients with food allergy, defined as prior history of symptoms consistent with IgE-mediated reaction to food(s) and positive skin prick testing (wheal ≥ 3 mm) or food-specific IgE > 0.35, were included. Demographics and clinical data were obtained from the electronic health record (EHR). Regression analyses examined the association between patient/guardian-reported primary language and healthcare utilization, and whether COVID-19 pandemic modified these associations.

**Results:**

Of the 1,418 patients with food allergy identified, 1,267 were primary English speakers and 151 were non-primary English speakers. Non-primary English-speaking patients had lower odds of EHR portal activation as compared to primary-English speakers (52.3% vs 81.8%, adjusted odds ratio = 0.43, 95% confidence interval (CI) 0.29–0.62). Regarding non-primary English-speakers, an increase in the probability of EHR portal activation (from 61.6% [95% CI 51.5% − 71.8%] to 70.0% [95% CI 60.8%−78.7%]) and food-related emergency department visits (from 3.7% [95% CI −0.4%−7.9%] to 19.4% [95% CI 10.7%−28.0%]) from the pre-COVID-19 to COVID-19 period was noted.

**Conclusion:**

Disparities in rates of EHR portal activation and emergency department utilization which were differentially affected by the COVID-19 pandemic were identified among non-primary English-speaking patients in a cohort of pediatric patients with food allergy at a tertiary care referral center in the United States. This study highlights the need for greater understanding of language-based health disparities in food allergy and for more equitable EHR portal access among non-primary English-speaking patients.

## Introduction

Food allergy (FA) is a significant public health challenge in the United States, affecting 8% of children and 10% of adults [1,2]. FA is associated with negative impact on quality of life and significant psychosocial burden for patients and caregivers [3–5]. Additionally, there are significant FA-associated economic and financial burdens including missed work, hospitalizations, emergency department visits, and expensive allergen-free foods [6]. Given the challenges in accessing subspecialist care and significant financial costs associated with FA, studies have identified disparities in diagnosis and management of FA among minority and socioeconomically disadvantaged patients [7–9]. However, little is known regarding potential health disparities among patients with FA who are non-primary English-speaking, despite the fact that over 21% of the U.S. population speak a language other than English (LOE) at home and over 8% of the population report speaking English less than very well [7,8]. Language is a known social driver of health: patients who speak LOE are more likely to experience healthcare access disparities and suboptimal health outcomes as compared to primary English-speaking patients in the US [9–15]. Given the sizeable proportion of the U.S. population who speak LOE, the potential for language-based health disparities should be evaluated in FA and targeted interventions should be developed to address these disparities.

Specifically in FA, language barriers may impact verbal and written communication with providers and impede understanding of written materials including medication labels or emergency action plans. Identification of safe foods may be impacted as food labels and menus are primarily listed in English only. Communication barriers are additionally compounded by socioeconomic disparities as individuals who speak LOE are more likely to have Medicaid insurance coverage, lower health literacy, and have lower incomes compared to primary-English speakers [16–18]. Healthcare disparities are well-described among patients with FA who are Medicaid-enrolled or of low socioeconomic status (SES). Medicaid-enrolled children were more likely to have delays in establishing allergy care, have underdiagnosis of FA, and low epinephrine autoinjector prescription fill rates [19,20]. Patients with FA who are from households of low SES are more likely to have food insecurity and have challenges accessing safe allergen-free foods, and greater likelihood of ED visits and hospitalizations for FA [21–23]. To our knowledge, no prior studies have assessed whether patients who speak LOE may have challenges with FA care and management. Prospective cohort studies assessing racial and ethnic health disparities in FA have previously excluded non-primary English-speaking patients [24,25].

Additionally, emerging studies revealed that the coronavirus disease 2019 (COVID-19) pandemic disproportionately affected patients who use LOE, including greater COVID-19 mortality rates in areas with higher proportion of people who use LOE and lower odds of telemedicine usage for subspecialty visits compared to primary English-speaking patients [26,27]. Specifically for patients with FA, there are few studies assessing the impact of COVID-19 pandemic and lockdowns impacting FA healthcare access. In a survey-based study administered to caregivers of FA patients, COVID-19 pandemic was associated with limited access to healthcare, increased food insecurity and increased overall stress, especially in households with incomes < $50,000 [28]. While this study by Pitt et al. focused on caregiver-reported experiences during COVID-19 pandemic, our study aims to add to the current literature by retrospectively assessing healthcare utilization of patients who use LOE and FA prior to and during COVID-19 pandemic. Specifically, there is limited literature assessing the disparities in the access of allergist evaluation and diagnostic testing, EHR patient portal or utilization of FA-associated emergency care among patients with FA and speak LOE. Understanding these disparities and how COVID-19 pandemic may have further exacerbated disparities in FA healthcare utilization, will help identify areas for quality improvement and intervention.

The primary aim of this study is to assess potential healthcare utilization disparities among non-primary English-speaking pediatric patients with FA seen at our institution. Our secondary aim is to explore whether COVID-19 pandemic further exacerbated disparities in healthcare utilization among non-primary English-speaking and primary English-speaking FA patients. We hypothesized that prior to the COVID-19 pandemic, food allergy healthcare utilization and outcomes disparities existed and were further amplified during the COVID-19 pandemic. Our retrospective study identified differential healthcare utilization and outcomes in FA patients in the pre- and during COVID-19 pandemic periods. Our findings offer new insights regarding the impact of language barriers on food allergy outcomes and identifies potential areas for intervention.

## Methods

We conducted a retrospective cohort study of children (18 years or younger) with a diagnosis of FA who were evaluated in the Yale pediatric allergy and immunology clinic between 1/1/2018-12/31/2021. The study was approved by the Yale University institutional review board under exempt determination (Protocol #2000027972).

Patients were initially identified by the Yale Joint Data Analytics Team if they had *International Classification of Diseases* (ICD)-9 or 10 diagnoses of FA (V15.0x, 995.6x; ICD10 codes Z91.10x, T78.0x). The electronic health record (EHR) was accessed for data collection by two authors (KW, NP) between September 1, 2022 – April 30, 2023 on secure computers to ensure each patient met inclusion criteria. Due to the nature of the medical record review, both authors had access to information that could identify individual patients. During the data collection process, each patient was assigned a coded number and the data of age, median household income of zip code, insurance type, self-identified race and ethnicity, primary language, clinic visit dates, allergy testing and outcomes, and number of and reason for ED visits were collected and inputted into REDCap electronic data capture tools hosted at Yale University, a secure web-based data management system [29,30]. No patient-identifying data such as name or medical record number was collected alongside data extraction. Only de-identified coded data were used during data analyses.

Using the methodology previously described by Mahdavinia et al., only patients with a convincing diagnosis of immunoglobulin E (IgE)-mediated food allergy–defined as prior history of symptoms consistent with IgE-mediated reaction to food(s) (i.e., immediate cutaneous, respiratory, gastrointestinal, or systemic reaction) and positive food-specific skin prick testing (wheal ≥ 3 mm) or food-specific IgE > 0.35 kU/L –were included [31]. Patients had to have at least 1 or more visits to the Yale pediatric allergy and immunology clinic between 1/1/2018-12/31/2021. Patients were excluded if they only had isolated oral allergy syndrome or had concomitant eosinophilic esophagitis or food protein-induced enterocolitis syndrome. These conditions were excluded as the study aimed to focus specifically on IgE-mediated food allergy healthcare utilization as non-IgE-mediated conditions such as eosinophilic esophagitis and food protein-induced enterocolitis syndrome may have associated emergency department visits for these conditions separately. Isolated oral allergy syndrome has low likelihood of systemic allergic reactions; as such, this would have a lower likelihood of emergency department visit than IgE-mediated food allergy. Patients with missing data were excluded from the analysis. Demographic data including age at initial and most recent visits in study period, insurance type (commercial, Medicaid, or self-pay), and clinic visit dates (including in-person and telehealth) were extracted from the electronic health record (EHR). As the study spans the start of the COVID-19 pandemic, we additionally categorized patients into pre-COVID-19 or during COVID-19 period if they had their initial clinic visit before the Connecticut stay-at-home order which was issued on March 23, 2020 [32]. Primary language was determined based on parent/guardian-reported preferred language, which is recorded during the first encounter for the patient at our institution and can be edited at any subsequent encounter. During the study period, none of the clinicians were fluent in any other languages aside from English. Video or phone interpreters were available for all visits. Race and ethnicity were self-identified in the EHR and were categorized in accordance with 2020 U.S. Census classifications [33]*.* Median incomes for the household zip code were obtained from the 2021 American Community Survey 5-year estimates [34]. Diagnoses of allergic rhinitis, eczema, and asthma were based on allergist documentation during the initial allergy visit during study period.

Outcome measures include EHR portal activation, allergy follow up visit, food-specific immunoglobulin E (IgE) testing, food-specific skin testing, oral food challenge, ED visit for food-associated reaction, and ED visit for food-associated anaphylaxis. Skin prick testing and oral food challenges were identified based on procedure billing codes (Current Procedural Terminology codes 95004 and 95076, respectively) and the outcome of the oral food challenges was obtained by manual chart review. During this study period, the general recommendation for patients with food allergies was to seek emergency care when anaphylaxis occurs and therefore ED visits for anaphylaxis can serve as a marker of accidental food exposures resulting in anaphylaxis [35]. Only ED visits associated with food reaction at our institution were included in the data. Reactions that occurred because of an in-office oral food challenge were excluded as these were not accidental food exposures. All clinician notes for ED visits associated with food reaction were reviewed by manual chart review to determine if the reaction met criteria for anaphylaxis. Anaphylaxis was defined according to the National Institute of Allergy and Infectious Disease and Food Allergy and Anaphylaxis Network criteria [36].

### Statistical analyses

We compared characteristics of primary English-speaking patients to non-primary English-speaking patients using either χ^2^ or Fisher exact test for categorical variables (sex, race, ethnicity, insurance, median household income quintile of zip code, atopic comorbidities, type of food allergy, number of food allergies, number of clinic visits attended, and if initial clinic visit occurred during COVID-19 pandemic) or Wilcoxon rank-sum test for continuous variables (age at initial or last clinic visit). Fisher exact test was used if frequencies were less than 5. Univariable and multivariable logistic regression was used for binary outcomes. Regression analyses were adjusted for *a priori* chosen variables which can affect the outcomes of interest independent of the primary exposure variable, and these covariates were age at initial visit, insurance type, median household income of zip code, and whether the initial visit was during the COVID-19 pandemic (S1 Table). Given that race and ethnicity and primary language are highly correlated with each other, we avoided including race and ethnicity as covariates in our model as multicollinearity may affect the reliability of the conclusions of the model since logistic regression models assume each covariate to be independent variables.

Results were summarized as unadjusted and adjusted odds ratios (OR, aOR) with surrounding 95% confidence intervals (95% CI) and estimated probabilities with 95% CIs. Since we did not conduct a statistical power analysis *a priori* and this was a retrospective study based on a convenience sample of patients which corresponded to the census of all clinic patients meeting the inclusion and exclusion criteria for the study period, there is potential for type II error. Therefore, due to the limited sample size and to reduce type II error, we reported notable findings at the two-sided alpha of 0.10 and focused on the 95% CIs covering clinically meaningful differences.

To further evaluate the effect of COVID-19 pandemic, we included the interaction term of primary language and initial visit during COVID-19 pandemic in separate logistic regression analyses of health utilization outcomes. We calculated the predictive margins of the estimated probabilities of health utilization outcomes for different combinations of primary language and if initial visit occurred during COVID-19 pandemic while holding all other covariates constant.

Statistical analyses were performed using Stata 18 (StataCorp. 2023. *Statistical Software: Release 18*. College Station, TX: StataCorp LLC).

## Results

### Study population

Among the 1,418 patients with FA that met our inclusion criteria, 1,267 (89.4%) were primary English speakers and 151 (10.6%) were non-primary English speakers (Table 1). Other primary languages included Spanish (8.4%), Portuguese (0.5%), Arabic (0.4%), Chinese (0.2%). No differences in age were observed between the two groups; the median age at initial visit in study period was 4 years old (range 1–9) and at last clinic visit was 5 years old (range 2–10) during the study period for both groups (Table 1). Both groups had higher proportions of male patients (57.6% and 55.0%, respectively). Most non-primary English speakers identified as Hispanic (76.2%) and other or not listed race (69.5%), whereas most primary English speakers identified as non-Hispanic (77.4%) and White (50.5%). Non-primary English speakers had higher rates of Medicaid coverage (85.4% vs 38.0%, p < 0.001) and greater rates of patients in the lowest quintile of median income of household zip code as compared to English-speaking patients (36.4% vs 17.5%, p < 0.001) (Table 1). Additionally, the food allergy profiles differed among the two groups as non-primary English-speakers had higher rates of egg, fish, and shellfish allergy. Primary English-speaking patients had higher rates of peanut, tree nut, and sesame allergy (Table 1).

**Table 1:** Demographics and clinical characteristics of pediatric patients with food allergy who were evaluated at the Yale Allergy & Immunology Clinic between 2018-2021.

	Primary English speaking (n = 1,267)	Non-primary English speaking (n = 151)	*P* value
Patient characteristics			
Sex, n (%)			0.50
Female	537 (42.4%)	68 (45.0%)	
Male	730 (57.6%)	83 (55.0%)	
Primary language, n (%)			
English	1,267 (89.4%)		
Spanish		119 (8.4%)	
Portuguese		7 (0.5%)	
Arabic		5 (0.4%)	
Chinese (Mandarin)		3 (0.2%)	
Other*		17 (1.2%)	
Median age, years (interquartile range)			
Age at first clinic visit	4 (1-9)	4 (1-9)	0.23
Age at last clinic visit	5 (2-10)	5 (2-10)	0.50
Race, n (%)			<0.001
Other/Not Listed	240 (18.9%)	105 (69.5%)	
White	640 (50.5%)	31 (20.5%)	
Black or African American	273 (21.6%)	3 (2.0%)	
Asian	107 (8.5%)	10 (6.6%)	
American Indian or Alaska Native	4 (0.3%)	2 (1.3%)	
Native Hawaiian or Other Pacific Islander	3 (0.24%)	0	
Ethnicity, n (%)			<0.001
Hispanic or Latino	231 (18.2%)	115 (76.2%)	
Non-Hispanic or Latino	980 (77.4%)	32 (21.2%)	
Not listed	56 (4.4%)	4 (2.65%)	
Insurance, n (%)			<0.001
Medicaid	482 (38.0%)	129 (85.4%)	
Commercial	763 (60.2%)	21 (13.9%)	
Self-pay	22 (1.74%)	1 (0.66%)	
Median household income of residential zip code (US dollars), n (%)**			
$28,008 - $55,000	222 (17.5%)	55 (36.4%)	<0.001
$55,001 - $89,744	460 (36.3%)	61 (40.4%)	
$89,745 - $149,131	463 (36.5%)	29 (19.2%)	
>$149,132	122 (9.6%)	6 (4.0%)	
Atopic comorbidities, n (%)			
Allergic rhinitis	476 (37.6%)	57 (37.8%)	0.97
Asthma	319 (25.2%)	31 (20.5%)	0.21
Eczema	579 (45.7%)	52 (34.4%)	0.01
Characteristics of food allergy			
Type of food allergy, n (%)			
Milk	127 (10%)	15 (10%)	1.00
Egg	360 (28.4%)	58 (38.4%)	0.01
Peanut	553 (43.6%)	44 (29.1%)	0.001
Tree nut	347 (27.4%)	27 (17.9%)	0.01
Fish	66 (5.2%)	23 (15.2%)	<0.001
Shellfish	82 (6.5%)	27 (17.9%)	<0.001
Wheat	21 (1.7%)	3 (2.0%)	0.74
Soy	18 (1.4%)	2 (1.3%)	1.00
Sesame	75 (5.9%)	3 (2.0%)	0.056
Other	41 (3.2%)	11 (7.3%)	0.02
Number of food allergies per patient, n (%)			0.04
1	941 (74.3%)	104 (68.9%)	
2	248 (19.6%)	32 (21.2%)	
3	59 (4.7%)	15 (9.9%)	
≥4	18 (1.4%)	0	
Total number of allergy and immunology visits attended			0.54
1	469 (37.0%)	59 (39.1%)	
2	326 (25.7%)	38 (25.2%)	
3	196 (15.5%)	26 (17.2%)	
≥4	276 (21.8%)	28 (18.5%)	
Initial clinic visit occurred during Covid-19 pandemic	583 (46.0%)	78 (51.7%)	0.19

*P* value reflects χ2 or Fisher exact test for categorical variables and Wilcoxon rank-sum test for continuous variables.

* Other languages in the cohort include Vietnamese, Albanian, Yiddish, Ukrainian, French, Korean, Russian, Pashto, Bengali, Laotian, and Tigrinya.

**Income quintiles from 2021 Census report on income. No median household income was below $28,008 and therefore the lowest quintile was excluded.

### Atopic comorbidities

Allergist-diagnosed atopic comorbidities differed among the two groups (Table 2). While the prevalence of allergic rhinitis was similar in both groups, non-primary English-speaking patients had lower odds of asthma diagnosis (adjusted odds ratio [aOR]=0.56, 95% CI 0.35–0.90) and eczema diagnosis (aOR=0.57, 95% CI 0.39–0.83), but a higher odds of multiple food allergies (aOR=1.49, 95% CI 1.01–2.19) as compared to primary-English speakers (Table 2).

**Table 2:** Univariable and multivariable logistic regression analyses of atopic comorbidities among English and Non-Primary English-Speaking patients.

Outcome	Primary Language (English, n = 1,267; non-English, n = 151)	N (% of subgroup)	Unadjusted		Adjusted*	
			OR (95% CI)	*P* value	OR (95% CI)	*P* value
Allergic rhinitis	English	476 (37.6%)	Reference		Reference	
	Non-English	57 (37.7%)	1.0 (0.71–1.43)	0.97	0.84 (0.57–1.25)	0.38
Asthma	English	319 (25.2%)	Reference		Reference	
	Non-English	31 (20.5%)	0.77 (0.51–1.16)	0.21	0.56 (0.35–0.90)	0.02
Eczema	English	579 (45.7%)	Reference		Reference	
	Non-English	52 (34.4%)	0.62 (0.44–0.89)	0.01	0.57 (0.39–0.83)	0.003
Multiple food allergies	English	325 (25.7%)	Reference		Reference	
	Non-English	47 (31.1%)	1.31 (0.91–1.89)	0.15	1.49 (1.01–2.19)	0.05

*Adjusted for insurance, median income of household zip code, age at initial visit, and whether initial visit was during Covid pandemic period

OR = odds ratio

95%CI = 95% Confidence Interval

### EHR portal activation and food allergy clinic healthcare utilization

Non-primary English-speaking patients had lower odds of EHR portal activation as compared to primary English speakers (52.3% vs 81.8%, aOR=0.43, 95% CI 0.29–0.62) (Table 3).

**Table 3:** Univariable and multivariable logistic regression analyses of EHR portal activation and food allergy clinic health utilization among English and Non-Primary English-Speaking patients.

Outcome	Primary Language (English, n = 1,267; non-English, n = 151)	N (% of subgroup)	Unadjusted		Adjusted*	
			OR (95% CI)	*P* value	OR (95% CI)	*P* value
Activated electronic health portal status	English	1,036 (81.8%)	Reference		Reference	
	Non-English	79 (52.3%)	0.24 (0.17–0.35)	<0.001	0.43 (0.29–0.62)	<0.001
At least 1 or more follow-up visit(s) to allergy clinic	English	796 (62.8%)	Reference		Reference	
	Non-English	92 (60.9%)	0.92 (0.65–1.30)	0.65	1.19 (0.81–1.75)	0.37
At least 1 or more oral food challenge(s) completed	English	258 (20.4%)	Reference		Reference	
	Non-English	27 (17.9%)	0.85 (0.55–1.32)	0.47	1.37 (0.84–2.24)	0.20
Food-specific IgE testing completed	English	961 (75.9%)	Reference		Reference	
	Non-English	114 (75.5%)	0.98 (0.66-1.45)	0.92	1.01 (0.66–1.55)	0.96
Food-specific skin testing completed	English	974 (76.9%)	Reference		Reference	
	Non-English	110 (72.8%)	0.81 (0.55–1.18)	0.27	1.0 (0.66–1.50)	0.99

*Adjusted for insurance, median income of household zip code, age at initial visit, and whether initial visit was during Covid pandemic period

OR = odds ratio

IgE = Immunoglobulin E

**Fig 1:**
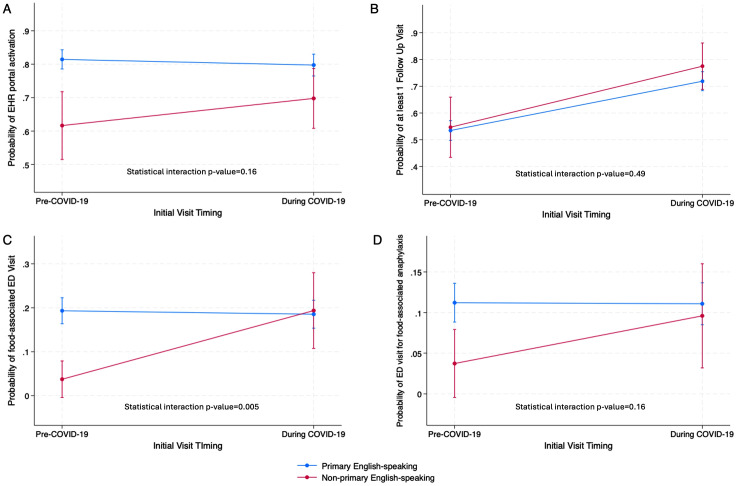
Adjusted estimated probabilities and 95% Confidence Intervals by whether initial visit occurred during COVID-19 pandemic (pre-COVID-19 vs. during COVID-19), from the logistic regression analyses for healthcare utilization.

Using the logistical regression model including the interaction term of primary language and initial visit during COVID-19 pandemic, the predicted probabilities of EHR portal activation from pre-COVID-19 to COVID-19 period remained relatively unchanged for primary English-speaking patients from 81.4% (95% CI 78.6% − 84.3%) to 79.7% (95% CI 76.5% − 83.0%) (average marginal effect p = 0.44) and increased for non-primary English-speaking patients from 61.6% (95% CI 51.5% − 71.8%) to 70.0% (95% CI 60.8%−78.7%) (average marginal effect p = 0.23) (Fig 1a).

Non-primary English-speaking patients had similar odds of attending at least one follow-up clinic visit as compared to primary English speakers (60.9% vs 62.8%, aOR=1.19, 95% CI 0.81–1.75) (Table 3).

Using the logistical regression model including the interaction term of primary language and initial visit during COVID-19 pandemic, both groups of patients experienced a notable and similar in magnitude uptick in follow-up visits. From pre-COVID-19 to the COVID-19 period, the predicted probabilities for follow-up visit increased for primary English-speaking patients from 53.5% (95% CI 49.8%−57.1%) to 72.0% (95% CI 68.3%− 75.4%) (average marginal effect p < 0.001) and for non-primary English-speaking patients from 54.7% (95% CI 43.5%−65.9%) to 77.5% (95% CI 68.9% − 86.2%) (average marginal effect p = 0.001) (Fig 1b).

**Fig 2:**
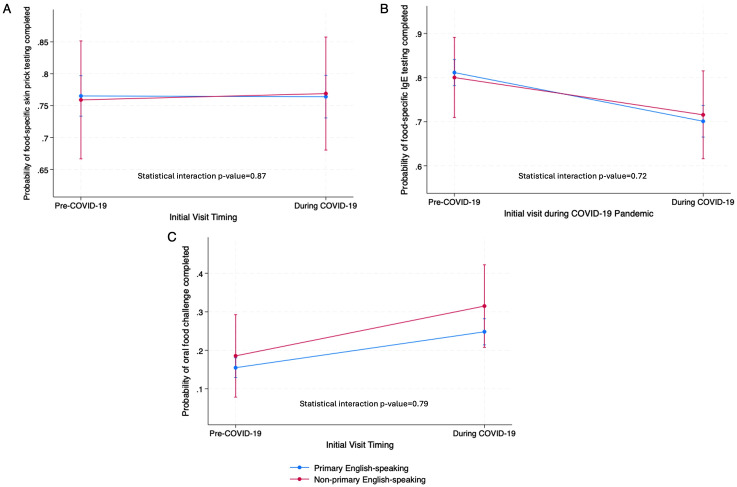
Adjusted estimated probabilities and 95% confidence intervals by whether initial visit occurred during COVID-19 pandemic (pre-COVID-19 vs. during COVID-19), from the logistic regression analyses for food allergy clinic utilization.

Compared to primary English-speaking patients, non-primary English-speaking patients had on average comparable odds of oral food challenge(s) completion (20.4% vs 17.9%, aOR=1.37, 95% CI 0.84–2.24), food-specific IgE testing (75.9% vs 75.5%, aOR=1.01, 95% CI 0.66–1.55), and food-specific skin testing (76.9% vs 72.8%, aOR=1.0, 95% CI 0.66–1.50) (Table 3). No between-group differences were observed by COVID-19 period (Fig 2).The predicted probabilities for oral food challenge completion increased for both primary English-speaking from 15.5% (95% CI 12.9% − 18.0%) to 24.8% (95% CI 21.4% −28.2%) (average marginal effect p < 0.001) and non-primary English-speaking patients from 18.5% (95% CI 7.8%−29.3%) to 31.5% (95% CI 20.8%−42.2%) (average marginal effect p = 0.085) from the pre-COVID-19 period to the COVID-19 period (Fig 2c). Food-specific IgE testing predicted probabilities decreased for primary English-speaking from 81.1% (95% CI 78.2%−84.1%) to 70.1% (95% CI 66.5% to 73.7%) (average marginal effect p < 0.001) and non-primary English-speaking patients from 80.0% (95% CI 70.9% − 89.1%) to 71.6% (95% CI 61.6% − 81.5%) (average marginal effect p = 0.21) (Fig 2b). Food-specific skin testing had no change across the pre-COVID-19 to COVID-19 periods for both groups (Fig 2a).

### Food-associated emergency department visits and anaphylaxis

Overall, non-primary English-speaking patients had lower odds of attending at least one food-associated ED visit (12.6% vs 18.8%, aOR=0.56, 95% CI 0.33–0.94) and food-associated anaphylaxis ED visit (7.3% vs 11.0%, aOR=0.57, 95% CI 0.30–1.11) as compared to primary English speakers (Table 4).

**Table 4:** Univariable and multivariable logistic regression analyses of food-associated emergency department visits and anaphylaxis among English and Non-Primary English-Speaking patients.

Outcome	Primary Language (English, n = 1,267; non-English, n = 151)	N (% of subgroup)	Unadjusted		Adjusted*	
			OR (95% CI)	*P* value	OR (95% CI)	*P* Value
At least 1 or more ED visit(s) for food-associated reaction	English	238 (18.8%)	Reference		Reference	
	Non-English	19 (12.6%)	0.62 (0.38–1.03)	0.06	0.56 (0.33–0.94)	0.03
**At least 1 or more ED visit(s) for food-associated anaphylaxis**	English	140 (11.0%)	Reference		Reference	
	Non-English	11 (7.3%)	0.63 (0.33–1.20)	0.16	0.57 (0.30–1.11)	0.10

*Adjusted for insurance, median income of household zip code, age at initial visit, and whether initial visit was during Covid pandemic period

OR = odds ratio

The COVID-19 period had a differential effect on the association between having at least one ED visit for food-associated reaction and primary language. From pre-COVD-19 period to the COVID-19 period, the predicted probabilities for at least one such ED visit increased from 3.7% (95% CI −0.4%−7.9%) to 19.4% (95% CI 10.7%−28.0%) (average marginal effect p = 0.001) for non-primary English-speaking patients, whereas it remained in the range of 19.3% (95% CI 16.3%−22.3%) to 18.5% (95% CI 15.3%−21.7%) (average marginal effect p = 0.723) among primary English-speaking patients (Fig 1c). Non-primary English-speaking patients also had lower predicted probability of patients with at least one ED visit for food-associated anaphylaxis during the pre-COVID-19 period (3.7%, 95% CI −0.5%−7.9%), as compared to English-speaking patients (11.2%, 95% CI 8.8%−13.6%), but this gap disappeared during COVID-19 as both groups had rates in the range of 9.6% (95% CI 3.2%−16.0%) (average marginal effect p = 0.13) to 11.1% (95% CI 8.5%−13.7%) (average marginal effect p = 0.94) (Fig 1d).

## Discussion

To our knowledge, this is the first study to assess the association of language barriers and the effect of the COVID-19 pandemic on FA healthcare utilization. We found that non-primary English-speaking patients overall had lower odds of EHR portal activation as compared to primary English-speaking patients and noted a differential effect of the COVID-19 pandemic between the two groups. Our findings suggest that disparities in EHR portal activation in our cohort existed prior to COVID-19 pandemic and while there was a trend towards an increase in activation rates during the COVID-19 pandemic, these disparities persisted into the COVID-19 pandemic. Accelerated by the COVID-19 pandemic, digital health and patient portals have become increasingly vital for healthcare access, patient engagement, and chronic disease management [37,38]. This study highlights the need for improvement in digital healthcare accessibility for non-primary English-speaking patients. Furthermore, our study found that non-primary English-speaking patients experienced sizeable increases in ED visits for both food-associated reaction and food-associated anaphylaxis during COVID-19 pandemic, while this differential effect was not observed among primary English-speaking patients.

Our finding of decreased odds of EHR portal activation is consistent with known disparities in EHR portal activation and use among non-primary English-speaking patients [11,39–41]. Our institution’s EHR portal is only available in English and Spanish, which is similar to many U.S. healthcare systems EHR portals as well [42,43]. Given 11 additional languages are spoken by our cohort, this likely contributed to lower odds of EHR portal activation. Bush et al. noted that non-primary English-speaking patients are less likely to be offered portal activation access in their cohort; while this was not assessed in our study, it is possible that similar disparities are present at our institution [44]. Compared to English-speaking patients, non-primary English-speaking patients are more likely to have inadequate internet and computer or smartphone devices which may limit portal access [40,41]. Lastly, challenges associated with low health and digital literacy, which has been described in greater association with non-primary English-speaking patients in prior studies, may make it more difficult to access EHR portals [40,41]. We observed a trend towards an increase in activation rates during the COVID-19 pandemic among non-primary English-speaking patients, similar to the findings of Kafashzadeh et al. The authors found that EHR portal activation increased after COVID-19 pandemic in their cohort of English and Spanish preferring patients with asthma, but Spanish preferring patients were still less likely to utilize the portal [11]. EHR portal use has been associated with improved health status awareness, increased adherence to therapies, improved quality of care; however, digital access disparities may disproportionately negatively affect non-primary English speaking patient outcomes [45]. Further studies investigating portal usage and engagement are needed, including assessing whether the increase in the EHR portal activation for non-primary English-speaking patients in our cohort has persisted into the post-COVID-19 era and whether disparities still remain. Areas of intervention to improve EHR portal access could be inclusion of more multilingual portal options and information technology support to assist with access. In-person support for enrollment and reference materials to support portal use in written and video format may improve digital accessibility [46].

In our study, non-primary English-speaking patients had overall lower odds of ED visit utilization for food-associated reaction. Previous studies have similarly found lower odds of ED utilization among non-primary English-speaking pediatric patients [47,48]. Notably, we found a differential increase in ED visits for food-associated reaction and anaphylaxis from the pre-COVID period to COVID-19 period which was not observed among the English-speaking counterparts. This differential increase in ED visits highlights a potential disparity given that pediatric ED visits were overall reduced during the COVID-19 pandemic [49]. It is possible that the differential increase may have been due to more accidental food exposures as a result of reduced allergen-free food access secondary to increased food insecurity during COVID-19 pandemic, which disproportionately affected households of lower SES [22,28,50,51]. Our cohort of patients who speak LOE was more likely to reside in zip codes with lower quintiles of median income and likely experienced worsened food insecurity during the COVID-19 pandemic. Contrastingly, allergy testing and odds of follow up did not differ in our cohort; it is less likely that delays in diagnostic testing or follow-up contributed to the increase in ED visits. While current literature regarding language-associated FA health disparities is limited, Correra-Agudelo et al. found racial differences in ED visits for food allergies and other atopic conditions, with Black patients having higher odds of ED visits during COVID-19 [52]. Outpatient healthcare utilization for patients seeking ED care was not assessed in this study so it is unknown whether the patients had comparable outpatient disease management [52]. Future studies will be needed to evaluate whether rates of ED visits continue to disproportionately affect non-primary English speakers in the post-COVID-19 era.

Compared to primary English-speaking patients in our cohort, non-primary English-speaking patients had overall similar or higher allergy utilization in the outpatient setting in terms of having a follow-up visit, oral food challenge, food allergy testing (i.e., skin prick testing and serum IgE) and were not differentially affected by COVID-19. The overall increase in probability of oral food challenges during COVID-19 pandemic may be due to an increase in number of allergists at our institution during COVID-19. Our findings also suggest that language barriers may not have negatively impacted follow-up rates or completion of diagnostic testing in our cohort. This may be due to the fact that our institution has reliable professional interpreter services available in-person, by phone or on video for both outpatient and inpatient care. Professional interpretation has been shown to be efficacious in reducing disparities in the quality of care for patients [53,54]. Further studies evaluating a broader population of food allergy patients who lack access to allergist or reliable interpreter services are needed.

Lastly, socioeconomic disparities present in our cohort of non-primary English-speaking patients (i.e., greater likelihood of Medicaid coverage and reside in zip codes with lower quintiles of median income) are important to note as these disparities are consistent with prior reports that show individuals who speak LOE are more likely to have Medicaid coverage and earn less than primary English-speaking individuals [16,17]. Consideration of all social drivers of health is crucial to ensure equitable healthcare for patients who are non-primary English speakers.

There are several limitations in our study: it is retrospective, limited to a single center, and encompassed a small cohort of non-primary English-speaking patients who primarily spoke Spanish. There is potential for selection bias as only patients with IgE-mediated food allergies who were seen in allergy clinics at an academic tertiary care medical center in an urban and suburban setting with reliable interpreter services were included; additionally, we excluded patients who had missing data or had eosinophilic esophagitis, FPIES, or isolated oral allergy syndrome which limits generalizability of the study. We may not have captured all FA patients in the electronic query, and we relied on provider documentation of clinical reactions to determine diagnoses of food allergy and anaphylaxis, which may have limitations in accuracy. Other confounders such as level of health literacy or English proficiency were not assessed as these data was not available in the retrospective review. Moreover, the covariates of insurance type and median household income of the zip code used in our data analysis are imperfect measures of socioeconomic status. Future prospective studies to better capture food allergy-associated clinical reactions, caregiver health literacy and demographics, and healthcare utilization are warranted. Studies involving patient and caregiver perspectives from diverse health literacy, cultural, and linguistic backgrounds will be important to better understand challenges in FA management and EHR portal use. Potential interventions to improve language-concordant care include increasing language accessibility of EHR portals, hiring more multilingual staff, and ensuring that all educational materials are available in multiple languages and at appropriate literacy levels.

In conclusion, disparities were identified in rates of EHR portal activation among non-primary English-speaking patients with FA as compared to primary English-speaking counterparts. Moreover, COVID-19 had a differential effect on non-primary English-speaking patients, leading to increase in probability of EHR portal activation and food-associated ED visits and anaphylaxis from pre-COVID-19 to during COVID-19. These disparities noted in our study support that language is a social driver of health [15]. Future efforts are needed to better understand language-based inequities in FA and inform patient-centered interventions to ensure equitable FA care.

## Supporting information

S1 Table Covariates used in logistic regression analysis. (DOCX)
