# Supplementary material for: Language barriers in pediatric food allergy care: A single-center study on healthcare disparities
Source: PLoS One. 2026 Apr 1;21(4):e0346248. doi: 10.1371/journal.pone.0346248 (PMC13042783; doi:10.1371/journal.pone.0346248)
Supplement: S1 Table — (DOCX) [file pone.0346248.s001.docx]

Supplementary Table 1. Covariates used in logistic regression analysis

| **Covariate** | **Definition** | **Data collected** | **Rationale for inclusion** |
| --- | --- | --- | --- |
| Age at initial visit | Age in years at the first pediatric allergy and immunology visit within the study period 1/1/2018-12/31/2021 | Continuous values (years) | Associated with outcomes |
| Insurance type | EHR-reported insurance type | Public, private, self-pay | Socioeconomic status, associated with outcomes |
| Median household income of zip code | Median income for the household zip code listed for the patient were determined from the 2021 American Community Survey 5-year estimates | Continuous (U.S. Dollars) | Socioeconomic status, associated with outcomes |
| Whether initial visit was during COVID-19 pandemic | If first pediatric allergy and immunology visit was after the Connecticut stay-at-home order which was issued on March 23, 2020, this was considered to be during the COVID-19 pandemic | Yes, No | Associated with outcomes |
